# Better quality of end-of-life care for persons with advanced dementia in nursing homes compared to hospitals: a Swedish national register study

**DOI:** 10.1186/s12904-020-00639-5

**Published:** 2020-08-26

**Authors:** Lisa Martinsson, Staffan Lundström, Johan Sundelöf

**Affiliations:** 1 Department of Radiation Sciences, Umeå University, SE 907 87 Umeå, Sweden; 2 Department of Palliative Medicine, Stockholms Sjukhem Foundation, SE 112 19 Stockholm, Sweden; 3 Department of Oncology-Pathology, Karolinska Institutet, SE 171 77 Stockholm, Sweden; 4 Betaniastiftelsen (non-profit organisation), SE 116 20 Stockholm, Sweden

**Keywords:** Dementia, Advanced dementia, Palliative care, End-of-life care, Hospitalisation, Gender, Age differences, Place of care

## Abstract

**Background:**

Hospitalisation of patients with advanced dementia is generally regarded as less preferable compared to care at home or in a nursing home. For patients with other diagnoses, young age has been associated with better end-of-life care. However, studies comparing the quality of palliative care for persons with advanced dementia in hospitals and nursing homes are scarce. The aim of this study was to investigate whether quality of end-of-life care for patients with dementia depends on age, gender and place of death.

**Methods:**

The Swedish Register of Palliative Care (SRPC) was used to identify patients who died from dementia in hospitals or nursing homes during a three-year period. The likelihood of death occurring at a hospital, based on age and gender differences, was calculated. Associations between 13 end-of-life care quality indicators collected from the SRPC and age, gender and place of care were examined in a logistic regression model.

**Results:**

Death at a hospital was associated with poorer quality of end-of-life care for 10 of the 13 measured outcomes when compared to death at a nursing home, and with better quality according to two of the outcomes. Death at a hospital was more common for men compared to women and for younger patients compared to older. Receiving fluids intravenously or via enteral tube in the last 24 h of life was strongly associated with death at a hospital. Women were more likely to have their oral health assessed and less likely to have pressure ulcers at death. Eight of 12 end-of-life care outcomes showed better results for the age group 65 to 84 years compared to those 85 years or older.

**Conclusions:**

Death in hospitals was associated with poorer quality of end-of-life care compared to death in nursing homes. Our data support the importance of advance care planning and individual assessments in nursing homes to avoid referral to hospitals during end of life. Despite established recommendations to avoid hospitalisation if possible, there were strong associations between younger age, male gender and hospitalisation in the end of life. Further studies are needed to investigate the role of socioeconomic factors in end-of-life care for this patient group.

## Background

The white paper on dementia care from the European Association for Palliative Care concludes that “transfer to the hospital and the associated risks and benefits should be considered prudently in relation to the care goals and taking into account also the stage of the dementia” [1]. Despite these recommendations, hospitalisation of patients with advanced dementia is common [2].

A study by Mitchell et al. of predictors and risk factors associated with end-of-life stage in dementia clearly demonstrates the difficulties in identifying this stage [3]. In a Swedish study, dementia was a predictor for readmission to hospital within 90 days of discharge [4]. In a Belgian study, 20% of patients with dementia were hospitalised during the last month of life, and slightly fewer than 5% were cared for in an intensive care unit during the last month [5]. Pneumonia, febrile episodes, and eating problems are common for advanced dementia and are associated with high six-month mortality rates [6].

It is difficult to know who will benefit from hospitalisation and who will not. There is evidence that people with dementia can experience a range of adverse outcomes in hospitals; they are hospitalised longer, and more often readmitted to hospital within 3 months, compared to patients without dementia [7]. For patients dying from dementia-related causes, death at a hospital has been shown to be more common for men, married compared to non-married people, and patients 80 years or younger, compared to older patients [8]. According to Sampson et al., patients with dementia in hospitals were less often prescribed symptom management medication and less often admitted to palliative care compared to hospitalised patients without dementia [9].

Old age (regardless of diagnosis) seems to be a factor that influences the palliative care content, regarding both place of death and utilisation of care. Gielen et al. showed that older persons were more likely to die in a nursing home, less likely to be transferred between care settings and stayed fewer days in hospital [10]. Old age has been associated with a lower chance of receiving specialised palliative care [11]. In a Danish population study, women compared to men and patients less than 40 years old compared to 80 years and older were more likely to be admitted to specialised palliative care [12].

Some studies have shown that age has an impact on palliative care for patients with cancer: younger patients with cancer were more likely to receive specialised palliative care in Spain and Italy [13], old age was associated with higher probability of death in hospital [14], a study in Canada on women with breast cancer showed that young age was associated with lower probability of receiving a palliative care–oriented model of health care services [15] and older people who died of colorectal cancer had a lower chance of receiving a palliative care programme [16]. A study using data from the Swedish Register of Palliative Care (SRPC) showed that younger patients with cancer were more often informed about their disease; evaluated for pain and other symptoms; and prescribed medications as needed against pain, anxiety and nausea. Younger patients also more often received palliative care consultation, and their next of kin more often received information and were offered support after death of the patient, compared to those of older patients with cancer [17].

Studies comparing the quality of palliative care for persons with advanced dementia in hospitals and nursing homes are scarce.

### Aim

The aim of this study was to investigate whether quality of end-of-life care for patients with dementia depends on age, gender and place of death.

## Methods

### Planning and setting

The SRPC collects data about quality of end-of-life care in Sweden using an end-of-life questionnaire (ELQ) [18]. The SRPC also contains data from the Swedish Cause of Death Register, which includes International Classification of Diseases 10 (ICD-10) codes describing underlying cause of death.

The SRPC was used to identify all patients who had died an expected death from dementia in hospitals (wards without palliative specialisation) and nursing homes (long- or short-term stay) during a three-year period from March 2012 to February 2015 and who had been reported to the SRPC. Death from dementia was defined as one of the ICD-10 codes F01, F02, F03 and G30, including subgroups, as the underlying cause of death. The study group was a subgroup of a larger cohort of patients who had died from dementia or cancer regardless of place of death, previously examined by the authors [19].

### Data analysis

Patients were divided into three age groups (below 65 years, 65–84 and 85 and above). The limit of 65 years was chosen because this is the cut-off for early onset dementia [20] and also the Swedish retirement age, and a common age in Sweden to qualify for elderly care. The limit of 85 years has previously been used in the literature, for example, in the Leiden 85 plus study [21].

Distribution of age, gender and place of death (hospital or nursing home) for the study group was described. The likelihood of death at a hospital, dependent on age group and gender, was examined using multivariable logistic regression with the oldest age group as reference. End-of-life care outcomes for 13 quality indicators available in the SRPC database were compared between hospitals and nursing homes using chi-square tests. Items in the database about symptom occurrence and relief were not used, because of their limited validity [22]. The outcomes were then analysed with a multivariable logistic regression model with age, gender and place of death (hospital or nursing home) as independent variables. *P*-values below 0.05 were considered significant. Patients without next of kin were excluded when analysing the outcomes regarding next of kin. “Don’t know” answers in the ELQ were considered missing data.

## Results

A total of 18,610 adults who had died from dementia in hospitals or nursing homes were identified in the SRPC database during the study period. Out of these, 1397 persons were reported to have died unexpectedly, and 365 persons had “Don’t know” reported regarding expected or unexpected death, resulting in missing data, and those were excluded. A total of 16,462 patients, 4752 with Alzheimer’s disease and 11,710 with other or unspecified dementia, remained in the study. A smaller proportion, 967 patients (5.9%), had died at a hospital, and 15,495 patients (94.1%) had died in a nursing home. The mean age for the hospital group was 85.6 years (median 86, range 55–101). The mean age for the nursing home group was 87.4 (median 88, range 41–110). Death at a hospital compared to death in a nursing home was more likely for men than women and more likely in patients aged 65 to 84 years compared to patients 85 years and above (Table 1).

**Table 1:** Numbers and proportions of death at hospital per age and gender category, and likelihoods of death at hospital compared to nursing home, depending on age group and gender. Odds ratios (OR) with 95% confidence intervals (CI) and *p*-values are reported

	Hospital deaths/all deaths (n)	Hospital deaths/all deaths (%)	OR	95% CI	*p*
Age groups in years					
41–64	8/95	8.4	1.436	0.688–2.995	0.335
65–84	418/5112	8.2	1.528	1.335–1.748	< 0.001
85–110	541/11,255	4.8	Ref^a^		
Gender					
Women	432/11,120	3.9	0.387	0.338–0.442	< 0.001
Men	535/5342	10.0	Ref^a^		

^a^ Reference

### End-of-life care outcomes in different care settings

When not adjusting for age or gender, 10 of the 13 measured end-of-life care outcomes showed better outcomes in nursing homes compared to hospitals, while 2 outcomes were better in the hospital group (Table 2). Consultations of specialised palliative care teams were uncommon. The patients were more likely to have a documented decision to shift to end-of-life care in nursing homes as compared to hospitals. Giving information to next of kin about the transition to end-of-life care was more common in hospitals, but offering a follow-up talk to next of kin after death of the patient was more common in nursing homes.

**Table 2:** End-of-life care outcomes for 13 quality indicators in hospitals and nursing homes. Differences in proportions between hospitals and nursing homes are calculated with chi-square test

Quality indicators	Missing (n)	Hospital		Nursing home		*p*
		n	%	n	%	
Specialised palliative care team consulted	0	22/967	2.3	192/15,495	1.2	0.006
Documented decision to shift to end-of-life care	1754	731/897	81.5	12,460/13,811	90.2	< 0.001
Next of kin given information about transition to end-of-life care	1284	714/878	81.3	10,215/14,300	71.4	< 0.001
Next of kin offered a follow-up talk after death of the patient	2968	263/617	42.6	9697/12,877	75.3	< 0.001
Pain assessed and documented during the last week of life	619	140/861	16.3	4838/14,982	32.3	< 0.001
Symptoms other than pain assessed during the last week of life	853	79/802	9.9	2773/14,807	18.7	< 0.001
Oral health assessed during the last week of life	1280	630/824	76.5	10,992/14,358	76.6	0.947
Prescription of PRN drugs against pain	62	839/949	88.4	14,461/15,451	93.6	< 0.001
Prescription of PRN drugs against nausea	225	398/927	42.9	9606/15,310	62.7	< 0.001
Prescription of PRN drugs against anxiety	133	705/939	75.1	13,171/15,390	85.6	< 0.001
Pressure ulcer at death	157	219/908	24.1	2278/15,397	14.8	< 0.001
Someone present at the moment of death	291	652/936	69.7	13,711/15,235	90.0	< 0.001
Fluids via enteral tube or intravenously during last 24 h of life	55	344/943	36.5	220/15,464	1.4	< 0.001

Patients who died in nursing homes were more likely to be assessed for pain and other symptoms during the last week of life, to be prescribed PRN drugs against pain, nausea and anxiety, and to have someone present at the moment of death compared to patients who died in hospitals. Oral health assessments were equally common in hospitals and nursing homes. Pressure ulcers at the time of death were more common in hospitals. Fluids given via enteral tube or intravenously during the last 24 h of life were much more common in hospitals (36.5%) than in nursing homes (1.4%).

### Age and gender differences

In the final model adjusted for gender, age groups and place of death, few gender differences remained. Women were more likely to have their oral health assessed and were less likely to have pressure ulcers at death compared to men. For the other 11 end-of-life care outcomes, no gender differences were found (Table 3).

**Table 3:** Logistic regression model comparing 12 end-of-life care outcomes for three age groups (in years) and for men and women, adjusted for place of death (hospital/nursing home). Odds ratios (OR) and 95% confidence intervals (CI) are reported

	n	%	OR	95% CI	*p*
PRN drugs against pain					
Age					
41–64	88/95	92.6	1.006	0.464–2.185	0.987
65–84	4799/5086	94.4	1.335	1.159–1.537	< 0.001
85–110	10,413/11,219	92.8	Ref^a^		
Gender					
Women	10,342/11,081	93.3	1.006	0.880–1.149	0.931
Men	4958/5319	93.2	Ref^a^		
PRN drugs against nausea					
Age					
41–64	59/94	62.8	1.108	0.726–1.691	0.636
65–84	3131/5037	62.2	1.069	0.997–1.147	0.060
85–110	6814/11,106	61.4	Ref^a^		
Gender					
Women	6832/10,984	62.2	1.038	0.969–1.113	0.285
Men	3172/5253	60.4	Ref^a^		
PRN drugs against anxiety					
Age					
41–64	79/94	84.0	0.991	0.568–1.730	0.975
65–84	4342/5063	85.8	1.127	1.024–1.240	0.014
85–110	9455/11,172	84.6	Ref^a^		
Gender					
Women	9405/11,041	85.2	1.017	0.926–1.116	0.728
Men	4471/5288	84.5	Ref^a^		
Pain assessed and documented during the last week of life					
Age					
41–64	29/93	31.2	1.025	0.658–1.595	0.914
65–84	1550/4916	31.5	1.039	0.965–1.118	0.313
85–110	3399/10,834	31.4	Ref^a^		
Gender					
Women	3428/10,726	32.0	1.042	0.968–1.121	0.277
Men	1550/5117	30.3	Ref^a^		
Symptoms other than pain assessed during the last week of life					
Age					
41–64	22/87	25.3	1.572	0.966–2.560	0.069
65–84	890/4830	18.4	1.049	0.960–1.146	0.294
85–110	1940/10,692	18.1	Ref^a^		
Gender					
Women	1984/10,584	18.7	1.080	0.988–1.181	0.091
Men	868/5025	17.3	Ref^a^		
Oral health assessed during the last week of life					
Age					
41–64	78/93	83.9	1.712	0.983–2.981	0.058
65–84	3696/4730	78.1	1.162	1.069–1.263	< 0.001
85–110	7848/10,359	75.8	Ref^a^		
Gender					
Women	7923/10,286	77.0	1.112	1.025–1.206	0.010
Men	3699/4896	75.6	Ref^a^		
Next of kin informed about transition to end-of-life care					
Age					
41–64	72/90	80.0	1.630	0.970–2.739	0.065
65–84	3549/4733	75.0	1.224	1.131–1.325	< 0.001
85–110	7308/10,355	70.6	Ref^a^		
Gender					
Women	7331/10,274	71.4	0.959	0.887–1.036	0.289
Men	3598/4904	73.4	Ref^a^		
Next of kin offered a follow-up talk after death of the patient					
Age					
41–64	69/84	82.1	1.776	1.005–3.140	0.048
65–84	3119/4152	75.1	1.161	1.064–1.265	0.001
85–110	6772/9258	73.1	Ref^a^		
Gender					
Women	6828/9217	74.1	0.983	0.903–1.071	0.701
Men	3132/4277	73.2	Ref^a^		
Documented decision to shift to end-of-life care					
Age					
41–64	75/84	89.3	1.066	0.531–2.141	0.858
65–84	4145/4574	90.6	1.216	1.079–1.371	0.001
85–110	8971/10,050	89.3	Ref^a^		
Gender					
Women	8934/9924	90.0	1.083	0.966–1.215	0.171
Men	4257/4784	89.0	Ref^a^		
Specialised palliative care team consulted					
Age					
41–64	3/95	3.2	2.895	0.902–9.293	0.074
65–84	84/5112	1.6	1.461	1.103–1.937	0.008
85–110	127/11,255	1.1	Ref^a^		
Gender					
Women	147/11,120	1.3	1.181	0.877–1.590	0.274
Men	67/5342	1.3	Ref^a^		
Someone present at the moment of death					
Age					
41–64	85/93	91.4	1.505	0.720–3.147	0.277
65–84	4554/5043	90.3	1.340	1.197–1.501	< 0.001
85–110	9724/11,035	88.1	Ref^a^		
Gender					
Women	9691/10,915	88.8	0.904	0.811–1.008	0.070
Men	4672/5256	88.9	Ref^a^		
Pressure ulcers at death					
Age					
41–64	13/94	13.8	0.829	0.460–1.496	0.534
65–84	767/5055	15.2	0.943	0.859–1.036	0.221
85–110	1717/11,156	15.4	Ref^a^		
Gender					
Women	1613/11,031	14.6	0.876	0.799–0.960	0.005
Men	884/5274	16.8	Ref^a^		

^a^ Reference

In the final model, 8 out of 12 measured end-of-life care outcomes showed better outcomes for the age group of people 65 to 84 years old compared to those 85 years or older. Next of kin informed about transition to end-of-life care and next of kin offered a follow-up talk after death of the patient were the only two outcomes where the difference in outcome between the age groups was more than 5 percentage points. The other outcomes where the age group of 65 to 84 years showed small but significantly better outcomes compared to the oldest group were PRN drugs against pain, PRN drugs against anxiety, oral health assessed during the last week of life, documented decision to shift to end-of-life care, specialised palliative care team consulted and someone present at the moment of death. For the youngest age group (41 to 64 years), one outcome (next of kin offered a follow-up talk after death of the patient) was significantly higher than the oldest age group (Table 3).

Three end-of-life care outcomes (PRN drugs against nausea, pain assessed and documented during the last week of life and symptoms other than pain assessed during the last week of life) showed neither gender nor age differences (Table 3).

Because of the low number of patients receiving fluids via enteral tube or intravenously during last 24 h of life in nursing homes, only hospital deaths were included in the logistic regression model adjusted for age and gender. The model showed no age or gender differences regarding the probability of receiving fluids (Table 4).

**Table 4:** Logistic regression model comparing the probability of receiving fluids via enteral tube or intravenously during the last 24 h of life for three age groups (in years) and for men and women, including only hospital deaths, not adjusted for place of death. Odds ratios (OR) and 95% confidence intervals (CI) are reported

	n	%	OR	95% CI	*p*
Fluids via enteral tube or intravenously during the last 24 h of life					
Age					
41–64	5/8	62.5	2.715	0.641–11.498	0.175
65–84	140/406	34.5	0.856	0.653–1.124	0.264
85–110	199/529	37.6	Ref^a^		
Gender					
Women	145/419	34.6	0.851	0.650–1.115	0.241
Men	199/524	38.0	Ref^a^		

^a^ Reference

## Discussion

In this national register study of 16,462 adults who died an expected death from dementia in hospitals or nursing homes we have shown that death at a hospital was associated with poorer quality of end-of-life care compared to death at a nursing home for all but three of the measured outcomes. Also, place of death was shown to have a larger impact on end-of-life care outcomes for patients dying expectedly from dementia, compared to age and gender. To the best of our knowledge, this is the largest study so far investigating the quality of end-of-life care in advanced dementia in nursing homes and hospitals, and the only one examining a wide range of quality-related care outcomes.

There are recommendations to, if possible, avoid hospitalisation for patients with advanced dementia [1]. In addition acute settings are not suitable for persons with dementia [23]. Despite this, little is known about where patients with dementia wish to be cared for at the end of life. Also, their families’ opinions and views of the healthcare staff are not fully known [24]. However, carers may be more satisfied with the care given if the patient had died at home compared to having died in a hospital [25].

A marked difference between deaths in nursing homes and hospitals was that 30% died without someone present in hospitals, while the corresponding figure for nursing homes was 10%, although all deaths were reported as having been expected. It is unclear whether this difference is due to lack of resources or if it could be addressed with better routines and awareness of end-of-life care.

Also, there was a pronounced difference between hospitals and nursing homes concerning administration of fluids via enteral tube or intravenously during the last 24 h of life. Over one third of the patients who died an expected death in hospitals received fluids, compared with only 1.4% in nursing homes. According to the white paper on dementia care from the European Association for Palliative Care, permanent enteral tube nutrition may not be beneficial and should as a rule be avoided in dementia, and hydration is inappropriate in the dying phase [1]. The current Swedish national guidelines for palliative end-of-life care advise against routine administration of fluids intravenously or via enteral tubes for most patients who are expected to die within a short timeframe [26].

This study found strong associations between younger age, male gender and hospitalisation for patients dying an expected death from dementia. These findings are in line with the findings from Reyniers et al. [8] and also in line with Houttekier et al., who found that women and patients with higher age were more likely to die in nursing homes than in hospitals [27]. A possible explanation for male gender being a risk factor for hospitalisation could be that men are more often cared for by their spouse at home compared to women, resulting in hospitalisation in case of acute deterioration. Marital status has also been shown as a predictor for hospitalisation [8] but could not be examined in this study because the SRPC database does not contain this data.

Identifying the patient as being in the dying phase can be a key factor in avoiding hospitalisations that do not benefit the patient. Several prognostic tools for survival estimates have been published, but it still remains a challenge to identify the late palliative phase of dementia [3, 28, 29].

Advance care planning has been shown to improve consistency of care with the patients’ goals [30] and may be a way to reduce unwanted hospitalisations and increase the use of palliative care [30]. A do-not-hospitalise order has been shown to reduce the probability of acute hospitalisation [31], and advance directives have been shown to predict less time in hospital for patients with dementia [26]. In the SRPC database, there is no information about these types of orders or why the patient was sent to hospital. Also, advance directives are not legally binding in Sweden. Referral to hospital of a patient may be based on the conclusion that it will be better for the patient or that it is not possible to properly care for the patient in the home care setting or at the nursing home. If the aim is high quality end-of-life care, it may be better for the patient to be taken care of at the nursing home, as the staff know how to communicate and meet the needs of persons with severe dementia. This study demonstrates that a majority of patients with dementia were given end-of-life care in the nursing home, indicating that a majority of patients suffering from dementia can be managed in nursing homes during end of life.

A strength of our study is the large sample size and the population-based data. Despite a large sample size, the group of patients below 65 years of age who had died in a hospital was small, making it hard to draw further conclusions for this subgroup. In the future, it would be interesting to further examine which patients with a dementia diagnosis are hospitalised in the end of life and why. This could possibly be achieved by collecting data, including socioeconomic factors, from medical records and/or the patient registry at the National Board of Health and Welfare.

## Conclusions

Death in hospitals was associated with poorer quality of end-of-life care compared to death in nursing homes. Our data support the importance of advance care planning and individual assessments in nursing homes to avoid referral to hospitals during end of life. Despite established recommendations to avoid hospitalisation if possible, there were strong associations between younger age, male gender and hospitalisation in the end of life. Further studies are needed to investigate the role of socioeconomic factors in end-of-life care for this patient group.

## Data Availability

The dataset contains personally identifying information, such as personal identity numbers, and potentially identifying information, such as date of death, and therefore is subject to ethical and legal restrictions on public sharing. We cannot share the dataset, which is on individual level, because it is not permitted according to the laws that apply in Sweden.

## Abbreviations

CI: Confidence interval
ELQ: End-of-life questionnaire
ICD-10: International Classification of Diseases 10
OR: Odds ratio
PRN: Pro re nata
SRPC: Swedish Register of Palliative Care
